# An “*in vivo*” analysis of crafts practices and creativity—Why affordances provide a productive lens

**DOI:** 10.3389/fpsyg.2023.1127684

**Published:** 2023-08-04

**Authors:** Michael Kimmel, Camilla Groth

**Affiliations:** ^1^Cognitive Science Hub, University of Vienna, Vienna, Austria; ^2^Department of Visual and Performing Arts Education, Faculty of Humanities, Sports and Educational Science, University of South-Eastern Norway, Notodden, Norway

**Keywords:** affordances, ecological psychology, creativity, interactivity, material engagement, micro-genetic analysis, crafts

## Abstract

Scholars are increasingly recognizing that creativity is grounded in the active sensorimotor engagement with the environment and materiality. Affordances—recognizable pointers to action opportunities in the ecology—provide a helpful prism for analyzing how this happens. Creative practitioners, as they seek aesthetic opportunities or innovation, depend on their sensitivity toward potentialities in their action space. Presently, we apply a high-zoom lens to a crafts process, giving our *micro-genetic research design an affordance focus*. By investigating one of the authors, a ceramicist and a practitioner-researcher, through her process of making of a vase, we tracked how affordances are responded to, developed, shaped, invited or, where necessary, rejected, as the ceramicist “routes” her creative trajectory. Several insights emerge: (1) The ceramicist's decisions—initially about general directions, then about aesthetic details—unfold while engaging with the clay; they emerge in stepwise fashion, but with a holistic orientation. (2) Choosing among affordances requires parallel sensitivities to object functionality, aesthetics and creativity, as well as technical feasibility; adhering to the proper technical procedure that provides the very basis for creatively relevant affordances to later arise. (3) While the hands and eyes engage with short-lived affordances the ceramicist must keep in view higher-timescale affordances that ensure a good task progression for making a vase, and affordances for the material's overall “workability”. (4) The ceramicist typically relates to momentary affordances in light of expected as well as imagined others, to ensure a coherent end product. (5) Affordances contribute to material creativity in more ways than typically recognized in the literature. They range from serendipitous “finds” to options developed with a large degree of creative autonomy; affordances may also be indirectly invited and practitioners strategically change probability distributions as well as providing an enabling background for generative action. Thus, a crafts practitioner brings forth unconventional affordances through active engagement, using a mix of exploration, strategy, and imaginative potential. Affordance theorists err when stressing the possibility to just “find” creative options or that perceptual acuity is the sole skill.

## 1. Introduction

This study will analyze how an expert ceramicist orients toward affordances in a creative production process. It presents an *in-vivo* analysis of a clay throwing event from the inside perspective of one of the co-authors, the ceramicist and practitioner-researcher C.G. (hereafter: the ceramicist). Specifically, we will examine how a vase's shape and aesthetics are developed and which creativity related decisions are made while interacting with the material. Broadly speaking, the way in which this clay throwing event evolves can be expressed through Schön's (1992) idea of “reflective conversation with materials” and Malafouris' notion of “material engagement” (2013), as well as reflecting Ingold's (2010) critique of hylomorphism, the false notion that crafts processes happen through full pre-design in the mind.

We will use affordances as a theoretical prism to analyze this creative engagement process. The theory of affordances after Gibson (1977, 1979) offers a perspective for understanding the grounding of cognition in the ecology. It can capture how sensorimotor skills mediate the act of engaging with materials such as clay and allows us to identify which perceptual targets a skilled ceramicist keeps “on the radar” and how opportunities for action are noticed or actively created on this basis. Both scholars of making and of creative cognition have recently added the affordance concept to their analytic toolbox. The present contribution will try to extend and nuance this toolbox, with multiple stakeholder communities in mind. General cognitive scholarship can expect insights on different kinds of activity that are mediated through affordances, whereas scholars of crafts and creativity are provided with a contextual analysis of the micro-workings of material engagement that sheds light on the interplay of creative and technical aspects.

Although our case-study can build on a handful of related studies, we will look at the material making process in a more comprehensive and detailed manner than usual. By disentangling the various components of how a vase is made, a continuous flow of overlapping and simultaneous events, our aim will be to clarify when and how affordances drive the process forward, discuss how affordances interconnect across time and space, and identify different analytic layers involved in this complex form of expertise. As will be shown, a vase emerges from a trail of multiple affordances, which mediate creative decisions, aesthetic finessing, technical correcting or embracing errors, keeping the material in a workable state, and in tracking the task progression.

To contextualize our analysis we will begin by situating affordances in the recent ecological and interactive turn in cognitive science. Then we will explore the nexus between affordances and creativity, present our case-study, and draw conclusions about contributions of the affordance perspective to understanding crafts skills as well as creativity.

### 1.1. Origins of affordances

Affordances designate opportunities for action that can be detected in the environment by an organism by virtue of it being *attuned* to the former, as proposed by Gibson (1977, 1979). Prototypical examples could be actionable properties like the sittability of a chair, the graspability of a handle, or the drinkability of a liquid. The function affordances were posited for is to mediate between perception and action in a direct way.

Gibson believed that action options, for example in the study of visual perception, are directly accessible through information rich ambient patterns without higher mental mechanisms having to add much. So, perceiving the affordance structure of one's current action space (as well as potentially the broader context one is in) allows detecting optimal or less optimal ways of moving in space, handling objects and tools, or social engagement, as well as recognizing things that are not afforded at all for action. A central thrust of Gibson's argument is that the foundation of cognition is not the ability of a being to categorize or reason about things, but to respond to and make one's way through the environment.

Gibson and his followers wanted affordances to overcome dualisms between agent and environment and therefore define them through a specific relationality between, for example, a species and its ecology or, by extension, a cultural form of life and its cultural environment (Heft, 2007; Rietveld and Kiverstein, 2014). Affordances thus depend on an agent having specific properties which are necessary to detect them and act on them. Since an agent and the environment in that sense co-constitute each other, the two are also to be taken as a single unit of analysis.

Learning in this perspective can be explained as a function of increasing sensorimotor *attunement* to a specific niche and its structures. Many attempts at formalizing this relationality have been made by Gibson's followers since, for example by demonstrating that some affordances are body-scaled, e.g., a particular body size allows walking through doors of a particular maximal width or climbing steps of particular maximal height. These relations have been used to specify so-called control laws for a specific type of behavior (Warren, 2006). More recently especially research in sports has shown how affordances relate to highly domain-specific skills, such as detecting patterns of possible interplay with two other team members to configure an attack configuration in rugby (Passos et al., 2012).

### 1.2. New contexts in creativity research

Psychologists speak of creativity when something is valued (e.g., due to its functionality) and in some respect novel (Amabile, 1996; Sternberg and Lubart, 1998). In creativity research, affordances are presently beginning to inform the emphasis on active embodied engagement set by “post-mentalist” scholars. To qualify as creative, an action must be afforded in the ecology, but in addition must also be useful and to some extent out of the ordinary because it is not commonly noticed, hard to execute, or norm-violating (see below).

This growing interest in affordances sits well with increasingly influential concepts such as “distributed creativity” (Sawyer and DeZutter, 2009), “material engagement” (Malafouris, 2013), “material dialogues” (Brinck and Reddy, 2020), “correspondences” with materials and situations (Ingold, 2017), or “creative thinking” (Malafouris, 2014). These trends reject the dominant emphasis of traditional psychological theories to seek mental functions as the sole underpinnings of creativity (Sternberg and Lubart, 1998). Rather than thinking of creativity as an innate and static ability, they accord interaction with the ecology a key role. In other words, not every aspect of creativity is held to happen “in the head”. Malafouris (2013, p. 145f) sees creativity as “moment-to-moment improvisational thinking inside the world”, thus stressing the importance of action. This interaction and embodiment-oriented paradigm disagrees with traditional views on a central point: it recognizes that acting in the world and receiving feedback from the world operate integrally with creative cognition.^1^ The senses are not anymore seen as mere delivery system for a centralized thinking device, nor are action systems mere actuators of solutions that are just passed on from this device. This claim gives a fully embodied and physical character to earlier views which emphasize that creative processes involve recursive movements between exploration and idea generation (Finke et al., 1992; Ward et al., 1998) or “reflection in action” (Schön, 1991). There is a class of causal effects that emerge from recursively engaging with the ecology, i.e. relational and transactional effects (Vallée-Tourangeau, 2014) that arise when fully embodied agents interact with their ecology. Similar ideas have been circulating since the 1990s in “4E” (embodied, enactive, embedded, and extended) cognitive science and are now being picked up by scholars from that community who study creativity (Davis et al., 2015; Malinin, 2016, 2019).^2^

In this context, affordances provide researchers with a concept to understand what creative guidance is to be found in the external world, i.e. why the material context is not just “a passive container or at best modifier of innovative action” (Yahklef and Rietveld, 2019). Since sensorimotor functions are mediated by ecologically available information, affordances are a key focus for studying creative process *qua* material engagement. Creative activities crucially depend on a person's sensitivity toward sensorimotor potentialities perceived in their action space, and possibly sensorimotor skills to invite these. The affordance perspective directs our focus to how agents become attuned to their particular ecology in order to perceive such “actionable” possibilities that either allow direct creative realizations or indicate steps toward a creative outcome. An affordance focus puts us into a position to ask which aspects of an information landscape creative persons pay attention to or explore, but also draws attention to skills for creating or “inviting” affordances.

Meanwhile, the concept of affordances, whose fundamental role for analyzing skills is well recognized, has been too little adapted to creativity related inquiries. Ecological psychologists have mostly focused on everyday examples that Costall (2012) refers to as “canonical affordances”. These are familiar and related to well-defined tasks and norms. As a consequence, many things commonly said about such affordances may not fully apply in creative contexts. A related problem is that scholars typically focus on how existing affordances are perceived, but have scarcely asked how non-existing ones might be invented, created, or developed.

### 1.3. How affordances relate to creativity

As Glăveanu (2014b) emphasizes, creative affordances—we prefer to speak of *creativity related affordances*—arise when boundaries of the possible or socially accepted are pushed or negotiated. Many affordances are placed in the zone of what is done under some frequent intention or cultural norm. Yet, at the fringes of this zone many possible actions exist that are currently unperceived, that remain to be discovered, or that are norm-violating. As Glăveanu also emphasizes, some affordances are not in existence yet and need to be *invented* or “generated by the combination or transformation of basic (existing) potentials” (p. 219). Such affordances can emerge by combining mundane ones in unusual ways or by putting mundane ones into a new context that changes their significance for action. Thus, creativity related affordances point to something never tried or contextually atypical, and they may relate to yet inexistent entities that yet need to be developed.

Both the ability to discover and to create unconventional affordances can be crucial for creativity (Withagen and van der Kamp, 2018). For example, an affordance can point to a highly novel action “hidden” in the same information pointing to a familiar action. Costall (2012, p. 51) notes that “we are usually very effective in co-opting objects in non-standard ways into our ongoing activities, for example, catching a spider under an upturned glass”. Yahklef and Rietveld (2019) explain innovative behavior as sensitivity to non-salient affordances which the environment may be replete with. They emphasize spontaneous, but unconventional responsiveness to the environment, which is “partly constitutive of innovative action” instead of being a passive container for innovative action (p. 10). New skills or skill combinations can widen this responsiveness, as can engagement with new material spaces.^3^ This implies that the creative exploitation of affordances may benefit from new ways of perceiving, but also from new technical abilities.

Baber (2021), who writes on affordances in design processes from a radically embodied cognition perspective, defines “creativity as the opportunistic response to constraints” (p. 169), but also as a deliberate manipulation and probing of these constraints that is not mere trial-and-error. He proposes that affordances can be seen as points of stability in a coupled human-artefact-environment system, such that creativity arises when system constraints are selectively loosened so transitions to new affordances become available.

To contextualize affordances more broadly in creativity research Glăveanu (2014a,b) proposes a framework of “distributed creativity” in which actors, audiences, artifacts, actions and affordances are all parts of creative systems, that require being analyzed in their interplay. Glăveanu (2020) *affordance-perspective theory* additionally highlights how important wider contextual orientations are: “Creative action fundamentally depends on the development of perspectives from which new and unusual affordances are revealed” (p. 345).

It has been rightly said that affordances are dynamically responsive to continuous interaction (Chemero, 2009), an emphasis that dovetails with creativity scholars stressing the importance of temporally extended engagement. Fluid interactions with external resources produce shifting affordance configurations (Vallée-Tourangeau, 2014). Therefore, a person's creative intent can crystallize through continued engagement, when the evolving affordance constellation hints at new possibilities for action (Baber, 2015). Embodied probing, manipulation or perspective switches can render salient new affordance-specifying information or suggest new exploratory moves. These mechanisms of “fiddling” or “intelligent fumbling” (Kirsh, 2014) are related to the idea of “creative thinging” (Malafouris, 2014). Thus, from a creativity perspective, acting on or transforming the ecology is just as crucial as the ability to spot a little recognized affordance. In addition, many creative options depend on one's specific interaction history with the ecology. Particular activity trails may be needed to discover an affordance. Gaver (1991) speaks of *sequential affordances*, opportunities which, when acted on, reveal information about further affordances.

When applying the affordance notion to creativity epistemological issues require consideration. Reducing affordances to *givens* in the world is unhelpful in this context, a one-sided focus that may result from the realist leanings of ecological psychology. As Costall (2015, p. 51) appositely puts it, in traditional Gibsonian treatments users of affordances are “not makers or creators but recipients of already established meanings” and merely presented as “finders” of what already exists. Realism presents affordances, including unconventional and possibly yet unknown ones, as *existing* relations in the world, independent of whether they are perceived (or ever will be). Drawing attention to the problem, Shotter (1983) emphasizes that prior to performance of activity within the environment “what further action it may afford must remain to a degree uncertain” (p. 27). This position better accommodates constructivist researchers who tend to emphasize the fundamental non-determinism of creativity. Christensen (2002, p. 57) appositely describes the clashing viewpoints: “Realists ‘find' solutions, whereas constructivists ‘create' them”. So, novelty must, by definition, be out there to be found. Christensen recommends a middle position and suggests that creative process should be described as an “oscillation between the actual, and the possible and the impossible” (p. 88). In a partner paper to this one (Kimmel and Groth, 2023) we present a framework, which probes into ways of bringing together the best of the realist and constructivist worlds and which treats perception and directed creative engagement as complementary loci of skill.

## 2. Methodology

We now discuss the issue of how affordances can help empirical researchers analyze creative processes.

### 2.1. Micro-process analysis

Our qualitative methodology follows the general lines of process-oriented creativity research (Gruber, 1989), but adds a micro-genetic focus to this. Micro-genetic methods can “unpack” trajectories of skilled action in their details, and—in our present context—track how specific micro-actions or short-lived perceptual acts add up within a creative process. The approach applies a highly granular lens and places events, sometimes down to a sub-second scale, on a timeline.

Some popular variants of the micro-genetic method are purely observational: *Cognitive Event Analysis* (Steffensen, 2013; Steffensen et al., 2016) has demonstrated that embodied engagement plays causal role in problem solving and that exploration, manipulation, perspective change, as well as feedback stimulation scaffold cognition. Similarly, *Kinenoetic Analysis* embraces a pico-level focus on problem solving through embodied engagement in visual puzzles and tasks like Scrabble (Ross and Vallée-Tourangeau, 2021b). The other main flavor of micro-genetic method is phenomenological. It uses so-called first- and second person (i.e., dialogical) methods, which are capable of exploring tacit praxis knowledge and which have the benefit of including non-observable aspects such as body habits, subtle perceptions, as well as intentions, interest or motivations. Application examples include micro-genetic interviews done on creative partner dance (Kimmel et al., 2018; Kimmel and Hristova, 2021). In this kind of study, interviewees are supported in the recall of details through video-feedback (cf. Lyle, 2003) and/or facilitative techniques such as *Explication Interviewin*g (Vermersch, 1994; Petitmengin, 2006; Høffding and Martiny, 2016; Valenzuela-Moguillansky and Vásquez-Rosati, 2019). The latter is a method in which details of an experience can progressively unfold in consciousness through a mindfulness-based, and dialogically supported way of elicitation.

Importantly, there are also ways to combine observational and first-person aspects. For example, Glăveanu (2013) combines interviews and think-aloud data with a detailed analysis of the process via head-mounted cameras in a creative crafts setting, whereas Groth (2017) studied her experiential knowledge of clay throwing in an auto-ethnographic think-aloud study. The present study combined first-person data with a detailed reconstruction of the video timeline, combining a body mounted point-of-view camera and a tripod mounted video camera from 2 m distance. Thus, what the crafts practitioner said and what visibly happened at that moment could be interrelated. The data collection itself proceeded in two steps: As the making of the vase unfolded, the practitioner engaged in “thinking-aloud” and commented during small breaks. After the vase was done, the process was jointly reviewed with the support of *Explication Interviewing* techniques.

### 2.2. Using affordances to model behavior

Affordance analysis inherently points two ways, namely to the nature of the ecological situation and to the skills and abilities of the agent operating in it. We are inspired by Fajen et al. (2009, p. 89) who propose that affordances can provide “a functional semantics” for analyzing interaction processes. In our crafts context, such a semantics will mean creating descriptive categories that allow us to identify key points at which information in the material environment “shunts” or guides the making process. More specifically, it will require specifying ways in which perception and action are coupled to give rise to creative effects, mediated by affordances.

Glăveanu (2014b) rightly warns that creativity lies in perception, invention, or utilization of possibilities, and not in the affordances *per se*. Thus, we should clarify which analytic role affordances can play, and which not. In our view, affordances can focalize the analysis of cognition and creativity but cannot subsume all that needs to be said about temporally extended cycles of perception-action coupling, the relevant unit of analysis.

Firstly, a line needs to be drawn between affordances and actual actions or decisions, which are what a behavioral or phenomenological analysis aims at. An affordance specifies action potentials, not actions themselves. It may or may not be recognized and it may or may not be picked up on if recognized. Also, most situations offer multiple affordances (Kimmel, 2012; Rietveld and Kiverstein, 2014) so the question arises which of these are detected and which are selected—the latter moves issues of decision making into focus, i.e. why some affordances are preferred to others. In addition, not all affordances are equally usable; some need extra action to improve their utility and others the willingness to embrace risks.

Secondly, affordances cannot offer a complete explanation of agency. To act, an agent must also find the afforded possibility attractive, given a task context or exploratory orientation. Consequently, researchers need to capture which affordances are treated as *relevant in context*. Given the wealth of affordances, we notably cannot yet predict action intentionalities other than in the special case of “strong solicitations” (Dings, 2018). Many affordances simply remain unexploited since they do not make sense for a task or seem uninteresting in view of intentions or norms. We must therefore not neglect a person's intentions or strategies and strive to find ways of analyzing the *in-situ* interplay between intentions and affordances.

Thirdly, we need to pay close attention to the dynamics of the wider system that reshapes constellations, often very rapidly. Affordances change not only in response to both one's own actions, but also as a result of the autonomous material dynamics such as clay changing its texture over time, as in our example below.

Fourthly, we are not looking for only one kind of phenomenon. Affordances occur at different timescales and in different functions. Some refer to global tasks, while others are more local (van Dijk and Rietveld, 2020). Some mediate decisions whereas others are short-lived and modulate an ongoing action through feedback control while decisions are carried out (Kimmel, 2012). As we shall stress in Section 4, there are multiple, partly parallel ways in which creative action is mediated.

Given these many ways in which affordance mediated action manifests, the analysis that is to follow will draw on literature that provides functional qualifiers for affordances such as “sequential” or “micro” affordances. While such attributes should not suggest distinct mechanisms, they take a particular *contextual role* of an affordance into view. For example, speaking of a sequential affordance means to specify the causal relation to preceding and subsequent affordances in the same context. We should perhaps stress for readers with a quantitative background how much qualitative research draws strength from such contextual descriptions.

## 3. The making of a vase

We have selected a crafts context as an illustration, an activity which involves tangible performative and embodied aspects and which throws into relief material and tool related affordances. Our partner paper (Kimmel and Groth, 2023) additionally discusses a dance example, treating interpersonal affordances in analogous fashion. Before reporting our case-study and analyzing it through the lens of affordances it will help to introduce the nature of crafts practices and what kinds of creativity to expect here.

### 3.1. Crafting—A brief introduction

In craft domains a material such as metal or clay, or fibers such as wood, paper, twine, or textile are manipulated with the hands or tools, transforming the material's form in an additive or reductive fashion. This requires long training and the acquisition of manual techniques, perceptual abilities, and experiential knowledge of materials. Crafting requires a multi-stage process, in which the aim is to produce an aesthetic, artistic and/or functional object, similar to the fine arts, architecture or design. Actions must be performed in a requisite general order, with a constrained duration, and relative timing of each action. Crafting is thus a temporally extended pursuit which must make sense as a whole. This can been referred to as involving a necessary chain of operations (cf. Leroi-Gourhan, 1993; Roux, 2019). This chain of operations results from the physical logic and procedure of material transformation required for some broadly specified object type (a vase, a jug, a bowl, a plate, etc.).

Although this general type of outcome is usually intended in advance, the encounter with the material typically provides guidance for many decisions made in the process. Scholars have begun to take interest in “material conversations”, emphasizing a quasi-dialogic negotiation between the maker and the material (Brinck and Reddy, 2020). For example, textures of wood (Ingold, 2013) or entanglements of fibers can guide the maker (Aktaş, 2019), which presupposes understanding “the vitality of a specific material” (Mäkelä, 2016, p. 3). Thus, interesting affordances are found as the craftsperson interacts with the material. We will refer to this phenomenon as *material emergence*. The degree to which a maker exploits material emergence varies from each case to the next because some crafts processes can involve a certain amount of pre-design. What can be generalized about crafts is that possibilities are typically more narrowly constrained than in free improvisation domains like dance or music. The fact that soundness and functionality of the product matter imposes bounds on creative risk taking and experimentation. Sheer inventiveness is of but little value if the outcome fails to meet expected standards in form, e.g., if the object is deformed or breaks. Consequently, creativity lies in how some basic functionality is developed and enhanced through aesthetic features or other interesting non-standard properties. What we deem creative about crafts processes subsists in the technical fundamentals and refines them in novel ways. We shall later return to the question how technical aspects of affordances relate to aesthetic options.

Tool use is fundamental to crafts contexts. Many affordances of the material remain inaccessible unless one uses the right tools. There can even be several production stages that use different tools, possibly in different locations too. The chosen technology and tools affect possible affordances to a great deal. For example, everything created on a potter's wheel will be round. Access to a suitable range of affordances also benefits from a well-kept and –prepared workshop. Which affordances these are depends on the choice of materials. Any material defines what can be made, thus delimiting the range of affordances and making others salient (e.g., sensitive materials can be very beautiful, but also more delicate). Craft practitioners will use their rich experiential knowledge of material properties to prepare the material for its purpose so it will manifest the desired affordances.

### 3.2. Descriptive analysis of a clay throwing process

Complementing historical-archeological and anthropological scholarship of clay throwing techniques on a potter's wheel (Van den Leeuw, 1993; Gandon et al., 2013; Roux, 2019), we will now micro-analyze a pottery process with the intent to make a vase. Our specific focus is the first stage of vase-making, i.e., the forming part known as clay throwing. Since this process offers a rich space for studying creative process we deliberately bracket out the many additional possibilities of the “post-production” stages that expand the creative possibilities (surface manipulation and decoration, glazing, and firing). In our set up, the ceramicist's objective was to find an aesthetically appealing silhouette for the vase. Specifically, the task was to “find an interesting shape” in the process of engaging with the clay, rather than developing a particular design idea beforehand. We thus selected a relatively open task for study, albeit within the general constraints of vase making.

Let us consider some basics of clay-throwing: on the rotating potter's wheel, the ceramicist shapes the clay form through outward or inward pushing or pressing the clay down from above. The process begins by centering the clay on the wheel head, and then shaping it so that the next stages can ensue—making the clay hollow and then pulling up the sides. Low and broad forms of clay when starting will yield bowl- or plate-like shapes, whereas high, narrow initial shapes yield a good starting point for vases. Any intended form also needs to be structurally sound, meaning that the soft clay needs to be shaped so it can carry its own weight throughout the process, which excludes excessively high, wide, or very thin shapes, and it requires keeping a slightly thicker layer in the base that holds up the upper levels of clay.

**Figure 1:**
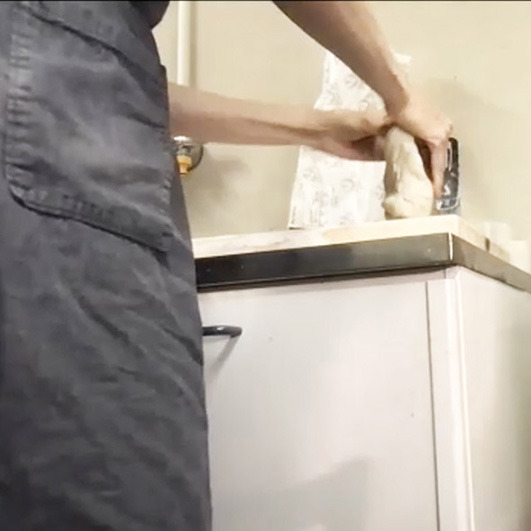
Preparation of the material on the plasterboard.

In our case, the ceramicist begins with choosing a basic task set-up for throwing clay. An informed decision is made regarding what type of clay is most suitable for making a vase on a throwing wheel. From the wide range of choices—ranging from coarse and stable clays strengthened with small chamotte particles to the very silky textured but un-plastic and precarious porcelain — she chooses porcelain. This choice adds an element of risk, but promises a fine and smooth surface that highlights even small nuances of shape, and turns to a translucent white when fired. The ceramicist then prepares the clay on a plasterboard through kneading in order to reduce air bubbles and eliminate excess moisture from the slightly too wet material (Figure 1). Her tactile evaluation indicates if the clay is too soft; if so it will not be able to carry its own weight later unless it is allowed to lose some moisture to the dry plasterboard. She also reports an expectation that more moisture will have to be added in the expected prolonged throwing process needed to search for an interesting shape; thus, only stiff enough clay can offset this. All these preparatory activities heighten the likelihood that desired affordances will appear and that undesirable material features such as air bubbles, a sagging clay body, or other indicators of “non-affordedness” remain absent.

**Figure 2:**
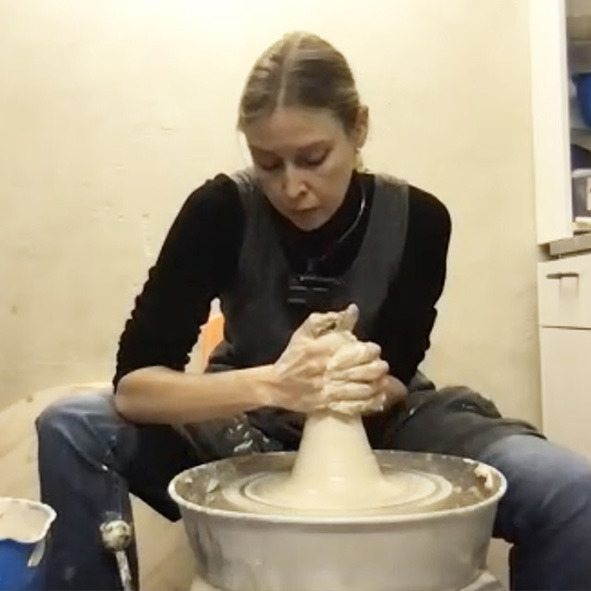
Centering the clay on the throwing wheel (repeated pushing up, pressing down).

Another preparatory step happens by centering the clay ball on the wheel. This is done by pulling the clay into a tower shape and pressing it down multiple times until it is perfectly centered (Figure 2). If the clay shakes in the hands of the practitioner this indicates a need for more precise centering. So material feedback provides clear indications of whether the degree of centeredness is at an *optimal* or moving toward a *critical point* (see Section 3.4). This tactile information specifies a micro-affordance for corrective feedback until the optimal point is approximated.

In view of the expected task of making a vase, the ceramicist decides to center the clay with a narrow base, as this will allow raising the form higher up. This initial decision gives rise to a (broadly defined) set of subsequent affordances. It also imposes certain action constraints and sensitivities on the next stages, as a tall hollow shape can easily get wobbly and difficult to balance. The process of raising the clay walls necessitates using water between the hands and the clay body to avoid the fingers getting stuck on the clay walls (and ripping the whole piece apart). However, using excess water also makes the clay suck up the water and so it quickly turns soft and unstable. The hazard is that soft walls, especially when they hang out- or inwards from the base, may collapse. With this in mind, the ceramicist tries to minimize the moistness, so that the required fine balance is constantly monitored. Raising the walls thus requires affordance-related brinkmanship (Kimmel and Rogler, 2018).

**Figure 3:**
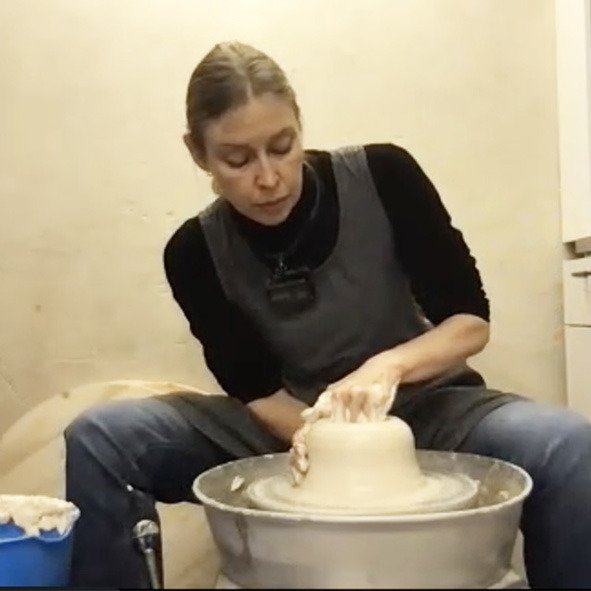
Making a hole and starting to prepare the base of the cylinder.

As the ceramicist searches for the middle point and starts making a hole at the center of the clay ball, she pushes her finger down, almost to the base (Figure 3). Again, this is done under prospective considerations of functional-technical kind, i.e., a kind of thinking ahead. The base should not be too thick or thin in relation to the other parts of the vase since this may cause cracks in the drying and in firing processes later. She also saves some extra clay in the lower portions of the base to support the future walls. Once the thickness of the base is felt to be appropriate the next action is to pull up the walls by pushing clay from the base upwards while pressing the fingers together from the inside and the outside simultaneously. The shape narrows and rises. Again, micro-affordances specify a critical range: the bottom of the wall must not get too thin; and the top rim of the clay must be kept straight and prevented from getting wobbly by using a controlling finger position or pressing a sponge toward it.

So far, the ceramicist has followed a “generic” strategy of creating as a straight a cylinder as possible to ensure the walls have an optimal thickness all the way around and will not risk sagging when manipulated later. This is meant to provide more creative shaping options later. The ceramicist says: “[by ascertaining what] the condition of the material is at that point, I will start to manipulate the shape to see if there are any starting points for what to do—what kind of shapes will come out of that”. This strategy provides conditions of possibility for yet unknown creative features later.

**Figure 4:**
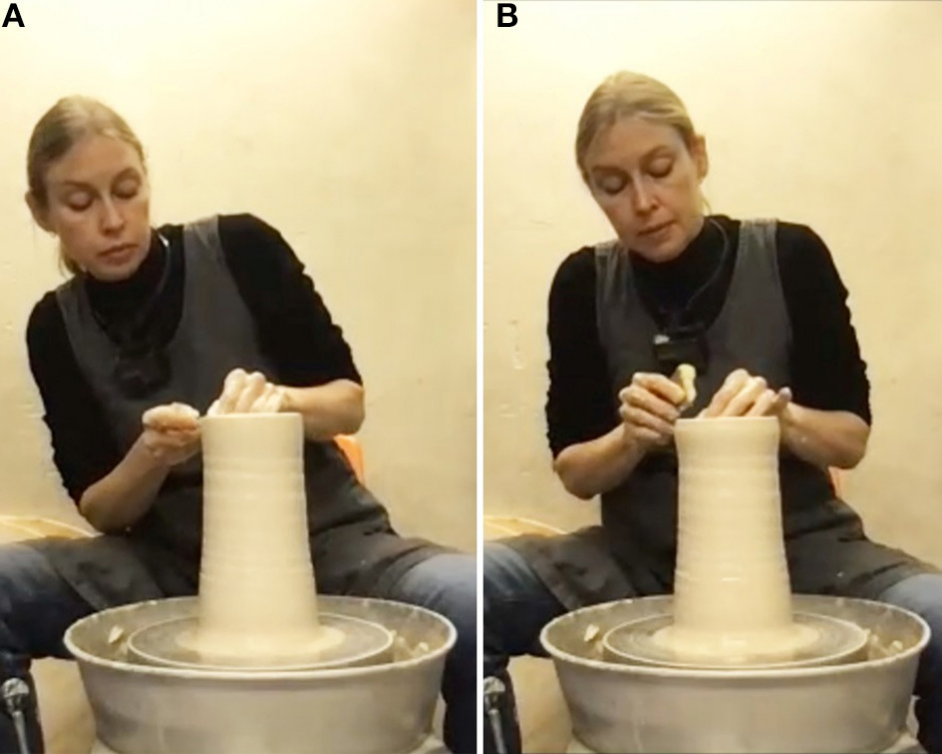
**(A)** The finished cylinder; **(B)** cutting off the top rim with a tool.

When the cylinder is about 25 cm high and its thickness is even from base to the top, this relatively uncreative basic part of the process comes to an end (Figure 4A). As she gets ready for the more creative part of the process the ceramicist finds one annoying element. She notices that the thick, uneven rim would look clumsy if left like that and applies a small correction by cutting off excess clay with a little stick. This is where her search for creatively interesting shaping options and features begins. In the process of correcting the rim, an unintended indentation emerges a few centimeters below (Figure 4B). The edge has slightly turned inwards, creating a bulging neck on the cylinder. This inspires a first creative decision. She considers the emergent neckline quite beautiful and decides to use the serendipitous “happy accident” (cf. Ross and Vallée-Tourangeau, 2021a) as a starting point: “I want to continue the shape based on these kinds of [wavy] movements. I also think it would be nice to have more of a body. Like more of an outgoing body to partner with the shape that goes up”. The undulating shape of the neck seems to suggest aesthetically relevant affordances for the rest of the object.

**Figure 5:**
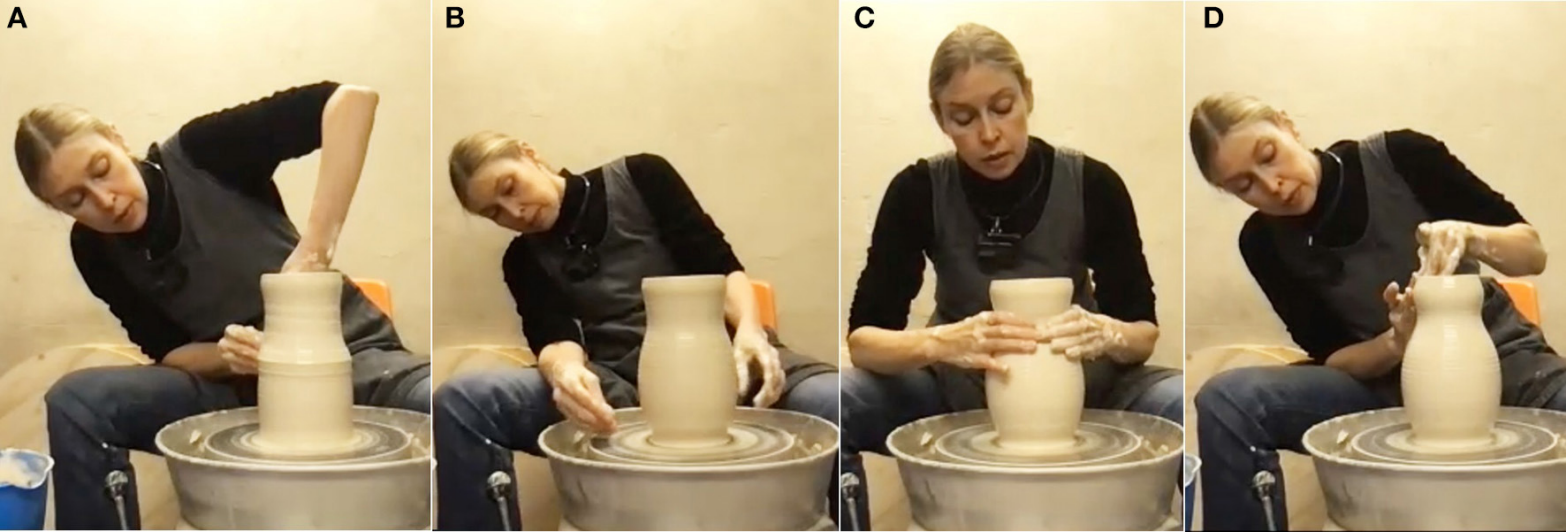
Working the middle: **(A, B)** shaping the cylinder into a vase; **(C, D)** a new curvature occurs (serendipitous moment).

The ceramicist's second major decision is an aesthetic one: to give the bottom parts of the cylinder a rounder body (Figures 5A, B). She pushes the wall outward from the inside in the middle section so it slightly bulges, while narrowing the shape of the lower base for contrast. In doing so, she stops and reports a perception of something aesthetically appealing emerging in the process: “even though I was first thinking that it needs to be narrowed down, [the concave curve at the base] actually mimics the curve up here” and is perceived as fitting the curvature of the neck's bottom as well as the concave of the upper neck. Another “happy accident” is accepted. At this point, actions on different parts of the object become highly interdependent. By narrowing the base more clay becomes available higher up and since the structure of the material “moves” throughout the object, working on the base changes the rim's shape further. The ceramicist now allocates attention to all the parts in their interplay. She balances the various sub-aims, prioritizes parts and considers which others to re-modify later.

The third aesthetic decision is to give the narrow and wide parts of the profile a stronger contrast. The ceramicist pushes out the “shoulders” of the vase by putting the hand inside the object. This creates a more bulging calabash-shape (Figures 5C, D) and immediately results in a slightly unexpected silhouette: “I thought I will just make it bulgy, but this line stops here and becomes a concave that then becomes round.” However, she likes what she sees and again accepts the serendipity. The overall lines of the object are described as a soft, round base then a straight line going outwards and then some roundedness, which turns into a concave vase neck and changes into convex again at the top. The underlying aim that guides this process is the ceramicist's wish to make the overall silhouette more pronounced. If the base isn't too thick and mimics the slim neck the parts are “communicating with each other and create more of a balance”. They are perceived as coherent—we might say a good gestalt is aimed at here.

**Figure 6:**
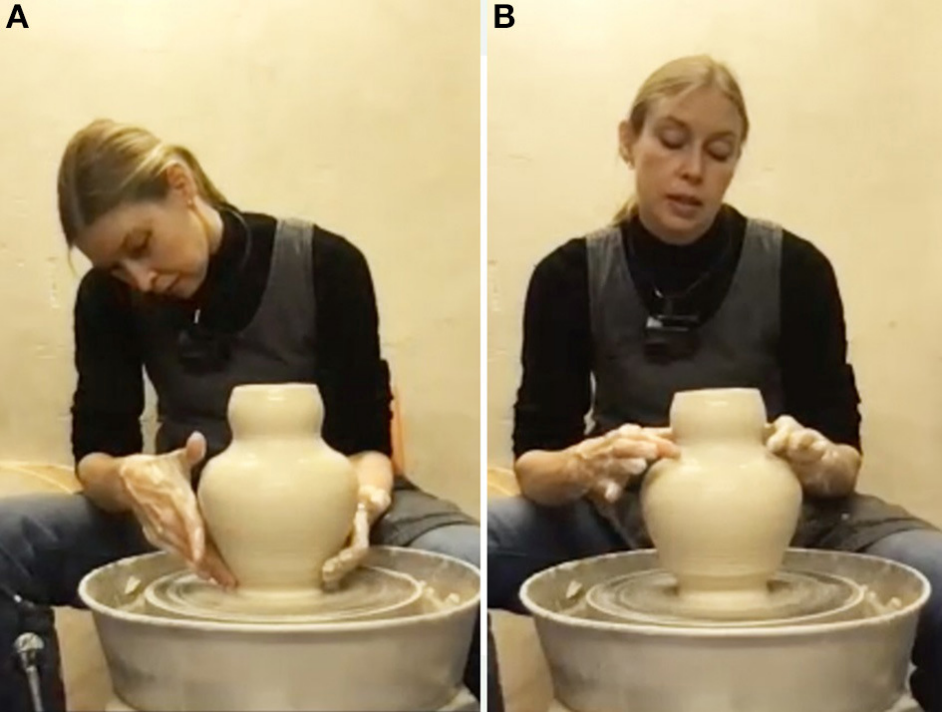
**(A)** An unexpected silhouette emerges; **(B)** correction (pushing back).

At this point the ceramicist sees and feels that the clay is getting very soft and prolonged further manipulation is likely to deform the shape. She is aware that a quick decision is needed and comments: “Otherwise, I will not succeed in getting to the end”. Although she reduces the risk a bit by eliminating moisture from inside the base with a sponge, the next manipulation confirms this expectation. As she manipulates the base, also the uppermost part of the vase is affected and opens up slightly again (Figure 6A). The ceramicist decides to make adjustments to restore its former shape (Figure 6B) as she dislikes the new more open rim-shape. After all, the previous shape was the initial inspiration for the whole vase. A potentially usable serendipitous affordance is deliberately counteracted because it does not fit the overall aesthetic idea.

**Figure 7:**
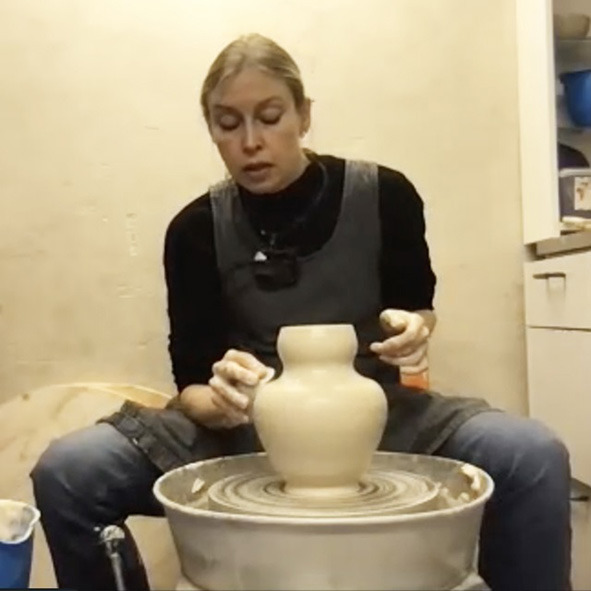
Finishing touches on surface; top part of vase turns wobbly.

Now, the actual shaping process is done. Although further intense manipulations are considered too risky, a short phase for adding the finer touches to the outward surface follows (Figure 7). Lines previously caused by using the fingers in the throwing process are smoothened with a metal trimming tool, which requires no further water to be added (this would not be afforded by hand). The ceramicist's reasoning here is that the lines would be too rough to fit the smooth surface and clash with the vase's general aesthetics. So again, an emergent affordance is rejected for aesthetic reasons, although it is perfectly possible in terms of functional considerations. Paradoxically, the shaping process is not actually done yet. During the final touches (of smoothening and of working on the neck) the very opposite of a serendipitous event occurs. The neck of the vase sinks a bit due to the overly soft clay, losing the “uplifting” shape the ceramicist wanted and the shape also becomes slightly wobbly. The clay's fatigue “has decided that we need to end this process” she says.

### 3.3. Summary of process

To recap, after building the basic clay cylinder, the creative “routing points” of the making process are mediated by specifiable affordances, mostly of a serendipitous kind. The creative arc developed out of the first aesthetic decisions whilst working on the rim when the byproduct of a technical correction imparted a shape-related inspiration for the rest of the project, namely to give the vase a wavy silhouette. The ceramicist explained this as follows: “this inside going curve was so tempting that I wanted to go around that avenue. And then make the rest of the silhouette fitting for that line.” This unintended by-product of prior manipulations could have been easily corrected, but instead the ceramicist “went with the flow” and accepted the serendipitous feature as an inspiration.

Later, another serendipity occurred when the bulge was added. The bulge produced a line of the body that seemed to “start a conversation” with the already existing rim. However, when the rim then started to lose its original shape due to manipulations of the base, it was restored in order to aesthetically balance the top and bottom parts. Therefore, this last emergent affordance (despite its sufficiently functional character) remained unused—it was not seen as a positive serendipity, but as being at cross-purposes with the overall aesthetic direction that had by now emerged.

It turns out that, although many “routing” decisions picked up on and amplified ongoing system dynamics, others counter-acted them to a degree. Hence, the momentary material dynamics often guided the ceramicist's actions, but not always. Emergent affordances and effects “coming for free” were rejected in favor of reworking if not considered in keeping with the aesthetic orientation.

### 3.4. Taking stock of affordance layers

Before we turn to creativity itself let us take stock of the different processes that involve affordances in this extended event and discuss in what aspects of the material they “live”.

Evidently, the ceramicist's know-how includes a great deal of previous experience with *basic material affordances* of different clay types and tools. She knows about the aesthetic quality of porcelain clay, but also its viscosity and susceptibility to softening and collapsing. Her basic choice delimits the possibility space for the specific project in terms of process duration and risks such as warping or de-centering that need to be calculated. It imposes basic parameters on the task. The material consistency of this clay type made itself shown throughout and influenced time management. Clay gets soft over time so the practitioner had to relate to warning signals and prioritize certain things over others before the time ran out.

During the clay throwing process the ceramicist oriented to *macro-scale affordances of evolving material state* for keeping the agent-ecology system in a balance. Moisture in the clay has a sweet spot and was haptically monitored to detect whether there was a lack or excess of water. Similarly, the object's orientation, whether it was still well-centered or if the shape was getting out of balance, was monitored both haptically and visually. Affordances at this level supported actions such as using a sponge to add or remove water or adjusting the speed of the wheel to counteract g-forces or gravity. Basic task parameters were hereby regulated.

Tracking *process affordances at a meso-scale* allowed the ceramicist to stay oriented to the requisite “chain of operations” of clay throwing, the procedural stages and task logic (correct order, transition timing, available duration, etc.). These were used to determine when the material was ready for the next stage and when a sub-task transition could be initiated. To do so, the ceramicist checked, for example, if the clay was stiff or centered enough to move on, but also evaluate the proportions, geometry and general shape, for example the height of the cylinder.

*Micro-scale affordances* supported the fashioning of aesthetic details and monitoring if technical basic standards were adhered to. They guided actions such as pulling up the walls or making them wider and allowed the fine-tuning as regards pressure, direction, intensity and positioning of the hands, pulling up or outwards, as well as operating the speed of the wheel to produce the right amount of g-force for the momentary action. These affordances are part of motor control through real-time feedback signatures that have optimal values and a critical range (Warren, 1984; Warren and Whang, 1987). Thus, micro-affordances point to error correction and modulations during critical moments, such as during the involuntary changes of the rim while manipulating the clay in the base.

To recap, the Explication Interview threw into relief that the ceramicist had to integrate multiple layers of affordance awareness, i.e., affordances used to track basic material qualities and to index how its “workability” was evolving, process-related affordances that signaled being “on track” in the chain of operations, and short-lived affordances that support proper technique and the development of aesthetic details. All this added to general knowledge of what can be done with the chosen type of clay which influenced the ceramicist's specific preparations, cost-benefit calculations, and risk awareness: the chosen material was expected to afford certain aesthetics, but also implied limited work-time and paying attention to material contingencies.

Thus, a crafts practitioner must *interdependently* regulate higher and lower timescale processes by keeping several affordance layers in view at all times. Notably, fast evolving manual techniques or explorations must always respect the global task requirements and keep material affordances of the long range, a favorable material milieu, intact. For example, the forming of the basic clay cylinder had to be kept steady as a structural requirement, even while smaller aspects of aesthetic surface finessing were happening. The theoretical implication is that expert practitioners must orient toward the evolving task ecology at multiple nested timescales at once, something frequently also seen outside crafts domains (Kimmel and Rogler, 2018).

### 3.5. How a creative whole arises

So what does the case-study specifically reveal about the relationship of affordances to creativity? Basically, crafts processes offer limited leeway for creative action, as functional and material constraints generate a narrow range of usable affordances. Creativity here typically means “walking on a knife's edge”, stretching the borders of possibilities toward novel options, and finding new ways to handle risk or fix problems. In traditional crafts in existence as long as wheel throwing most functional variants have already been discovered; hence the more interesting space for creative development is that of aesthetic choices.

In our case-study creative aspects entered at specific “routing points” of the process where the ceramicist made directional decisions about formal and aesthetic features. About five of these occurred, all in the second half of the making process (i.e., after the basic cylinder was complete). They all concerned the vase's silhouette. These creative routing points fall into three complementary categories: Some creative decisions that shape the overall trajectory emerged in the process without being deliberately created—they exemplify serendipity, i.e., “happy accidents” the ceramicist picked up on and accentuated further. Small deviations or by-products of technical operations led to aesthetically interesting affordances here.^4^ Other creative decisions reflected the practitioner's creative fancy and were deliberately implemented. While the former type just “found” an interesting affordance and “went with it”, the latter developed a creative constellation. A third frequent, but often unnoticed category of creative decisions concerns such that emerged from fixing or managing problems such as trimming the rim when it got wobbly (some more challenging ways of solving problems often become influential and are emulated among ceramicists).

Overall, both the accepted moments of serendipity and more deliberate ways of developing the vase concretized the aesthetic idea that had started to emerge when the accidental effect on the upper rim happened, a creative perspective which was progressively fleshed out. In terms of our above affordance timescales the creative routing decisions sat between the meso- and micro-scale. Some occurred at natural junctures where it is clear a new decision is needed. Others resulted when micro-scale affordances “spilled over” into larger creative decisions.

Over the various stages of making the vase the ceramicist worked out different aesthetical dimensions separately, which are distributed in space, but which need to make sense together in the end. Each of the micro-aesthetic features had to be technically afforded in itself, but they were interconnected. For example, parts of a clay object affected each other both in terms of functionality and aesthetics. Thus, the practitioner oriented toward micro-affordances of the clay object with a view toward the completed vase, the product. The vase's various features were orchestrated to cohere aesthetically. So, the creative aspect was, to a major extent, a gestalt effect, not an additive effect of individual actions on small affordances. This implies that the overall creativity depends little on individual affordances. Instead, each individual affordance was seen or imagined in light of possible others to produce coherent local actions that add up to a good gestalt. The practitioner thus related to multiple distributed affordances *holistically*.

### 3.6. Materiality and the imagination in interplay

While our affordance-based analysis highlights the sensorimotor aspect of craft skills, our data cautions against reducing the whole creative process to this. A genuine interplay of affordances with the imagination emerged in two spots of the Explication Interview:

We discussed a routing point in which a “happy accident” immediately triggered an autonomous ideation process in which other features in the lower portions of the vase were imagined to fit nicely with the curvature that emerged in the upper part. The ceramicist evoked an imagination about what the whole vase might look like in the end. All subsequent actions not only resulted from the perceived state of the clay object, but also from what was imagined in relation to it. The imagination imparted an overall vision about how features could be coherently developed. Anticipatory aspects of an imaginative kind similarly occurred when the ceramicist, based on her rich experience, “reasoned ahead” about implications of the chosen clay, tool requirements, and risks. Such foresight was needed to avoid “fighting” the material's constraints later and was crucial for an integral task management. The orientedness this bestowed in turn made particular further affordances salient. A third imaginative aspect not explicitly discussed above concerns how the ceramicist used visual-kinaesthetic imagery of basic elements and procedures as a resource (cf. Keller and Keller, 1999).

Evidently, such imaginations are materially grounded; they are triggered and constrained by the givens in the workspace. Yet, phenomenological evidence also suggests distinctive signatures: imaginations evoke absent details and the “not yet real”, remain open to revision and can be “holoptic” (i.e. co-actualizing hidden with perceptually present features), as well as allowing relatively unconstrained manipulation. For example, inspecting the vase from below is impossible while fixed to the wheel, but possible by rotating it in the imagination. Hence, material perceptions and their imagined complements have overlapping, but non-identical phenomenologies.

Given that crafts and design theory has for long emphasized design-driven processes—for reviews see Dorst and Dijkhuis (1995), Groth (2017)—a rapprochement of imagination scholarship with affordance scholarship is an important task for the future (Koukouti and Malafouris, 2020). Without falling into the extreme of *hylomorphic* theorizing, we must also recognize aesthetic strategies that draw resources from beyond the material situation: Petre et al. (2006) report that textile designers sometimes start from a class of aesthetic aims or from general design concepts and then looking for suitable means to implement these, or they try to re-situate abstracted patterns from other designs in the material context at hand. The situated imagination may also be infused with case-analogies or feature re-combination, and putatively even creativity mechanisms such as divergent thinking.

While these are empirical observations to take seriously, how to theoretically interpret the imagination within an ecological paradigm is currently debated: one influential view would define imaginings as affordances of the longer range (van Dijk and Rietveld, 2020), or as nested affordances (Rucińska, 2021), thus emphasizing their materially grounding. This approach aims to extend the non-representational foundations of Gibson's approach. Other views prefer to slot the imagination as a partner mechanism to affordances, notably Glăveanu (2020) who posits a dialectic between affordances and wider “perspectives” imparting creative orientation. The wisdom required to recognize the utility of a perceived serendipity is a similar partner mechanism reclaimed by creativity researchers Ross and Vallée-Tourangeau (2021a). Prima facie, both interpretations are compatible with an ecological perspective, where the former defends an inclusive view, and the latter sees affordances in a more partial capacity for explaining cognition (on their theoretical scope see Golonka and Wilson, 2019, Kimmel and Groth, 2023).

## 4. Discussion

We are now ready to draw out a number of general conclusions about the relationship between affordances, crafts process, and creativity. Several insights emerge about the “texture” of the extended creative processes in a material domain.

### 4.1. The integrality of skills

Somewhat counter-intuitively perhaps, crafts creativity builds on considerable standard know-how. A process referred to as creative as a whole is partly mediated by affordances that are non-creative, especially with regard to basic techniques and the process set-up. Creative affordances are typically considered relevant only late in the forming stage or in the post-production stages (unless unusual materials, tools, or techniques are additionally used). Thus, for creative affordances to appear in the first place, standard procedural constraints and the constraints of the chosen material must be adhered to. In crafts we cannot causally separate creative affordances from ordinary ones which “set the scene” and provide essential first anchor points for creative affordances to arise later.

The integrality of meaningful practice implies that affordances depend on a set of structured practices that precede or transcend the moment of action. The methodological implication is that we should hold this “periphery” of creativity in view in its causal contribution and not focus on creative moments in isolation. The same would, for example, be true in regard to applying standard science practices as the necessary ground for discoveries or even paradigm changes to occur.

This also indicates a need to talk about affordances with utmost context-sensitivity: we have seen that many in principle afforded actions scarcely make sense at a particular moment because they violate the time budget or the logic of process; retrospective and prospective considerations constrain the real-time “conversation” with a material. Likewise, affordances in the material are filtered through the crafts practitioner's interests and preferences where creative or aesthetic “visions” deselect many “raw” material options. What seems subjectively afforded in context is a question of greater analytic interest than talking about materials in a general way.

### 4.2. Multiple criteria of affordedness

One data interpretation challenge we faced was relating affordance theory to the multi-criterial field in which crafts practitioners operate. For them, multiple concurrent criteria coincide in a “good” affordance. The end product should combine soundness (*function*) and aesthetics (*beauty*), as well as, if so desired, a modicum of innovation or subjective novelty value (*creativity*). The functional orientation is quite basic, as a vase must neither collapse in the making nor break later. Practitioners integrate this with an aesthetic orientation, seeking a pleasing gestalt or interesting visual or haptic effect, as well as novelty. In addition, practitioners orient toward *technical and task-logic* criteria needed to realize all former aspects. Doing things in the right order and for the right amount of time guarantees functional soundness later.

A general observation from our case-study and others analyzed by us is that practitioners reason quite explicitly about how these criteria influence each other. The aesthetic compromise made toward the end when the clay got “tired” is representative of the fact that expert practitioners are acutely aware of the frequent need for compromise and prioritizing criteria. A similar compromise can be seen in the choice of porcelain, that has the benefit of translucency, hence aesthetics, but which comes at the cost of a limited flexibility of shapes and limited work-time. Practitioners are challenged to confidently handle these trade-offs, e.g., by choosing to embrace some technical risks for creative gains. More generally, much creativity is *brinkmanship* between the technically possible and the creatively or aesthetically desired. Technical risk taking can widen the possibility space, but at a cost: In another session we recorded, the ceramicist took an aesthetic risk, but the clay collapsed, resulting in a non-functional vase. Such risks can be partly, but never fully buffered through the proper preparation of the material such as through eliminating moisture, thus keeping more precarious affordances “alive”.

Furthermore, which material possibilities are deemed to be best afforded depends both on the practitioner's broader *perspective* as proposed by Glăveanu (2020) and the wider context (e.g., an installation artist will at times sacrifice functionality for supremely new formal features in ways a professional ceramicist cannot). In light of this, saying de-contextualized things like “clay affords kneading” is unhelpful. We cannot take momentary material states at face value because practitioners do not evaluate affordances in momentary terms only. Various factors filter among affordances that might be quite interesting in other projects. Affordances get deselected if they do not fit the current aesthetic preferences, if they clash with global task constraints or available time, or when they seem interesting but too difficult to realize. By a similar token, what seems *subjectively* afforded depends on the risk the practitioner is willing to take. Finally, technical or skill limits may determine what creative aspects can be exploited. Some creative affordances are easy to notice, but difficult to exploit, with easier to exploit ones often seeming mundane or unaesthetic.

### 4.3. Affordances, processuality, and emergence

A basic characteristic of crafts is that materials can be controlled to a relatively high degree (much more than, e.g., an improvising dance partner can be controlled). This allows creating complex affordance-bearing material constellations through the appropriate manipulations. Increasingly more demanding features can be produced by gradually bringing forth the full potential lying dormant in a material.

In this context, an interesting question to ask is what is expectable (or controllable) and what is contingent in a crafts process. It is true that the reported process was oriented toward a globally specified goal and object type, with a task progression that was expectable in its broad lines. Thus, raising the walls of a vase required a roughly defined sequence of action priors such as preparing, centering, and repeatedly moisturizing the material (a pot would begin similarly, but then begin to differ in the later stages). Hence, the wider *affordance landscape* (Bruineberg and Rietveld, 2014; Rietveld and Kiverstein, 2014) was expectable for the ceramicist. However, regarding aesthetic and creative details some of the more interesting affordances only emerged through the material engagement process itself. Thus, developing the vase's rim and bulge were shown to be emergent processes that unfold in a path-dependent manner over several stages.

On the one hand, the specific affordances that emerged depended on the contingent interaction history and subtle handling dynamics. The creatively most interesting affordances quite possibly resulted from highly context-specific details of how hands and clay “dialogued”. It is known that embodied interactions can come to have non-linear or otherwise multiplicative properties of a complex self-organizing system, a dynamic systems concept (cf. Baber et al., 2019). Of course, this was skilfully guided and constrained by the ceramicist, given that the many different ways of *managing emergence* in the agent-ecology system (Kimmel et al., 2018; Kimmel and van Alphen, 2022).

On the other hand, the ceramicist's intentional stance toward the process enabled all this in the first place. She initially decided to give leeway to *material emergence*, as she said: “I was trying to manipulate the shape as little as possible. And just go with what was emerging from that situation.” How much a practitioner “empowers” material emergence to bring forth interesting affordances depends. For example, the ceramicist commented that “if I wanted to make it teardrop shape, I could have done that consciously”. Someone intent on following a prior design idea could have easily blocked many serendipitous affordances through a stricter control of the clay. Thus, serendipity need not be exploited as frequently as in our case-study. Potentially creative chance affordances can be rejected in favor of specific design ideas or standard patterns.

### 4.4. Types of affordance-mediated activity

We would now like to turn to our objective of developing a semantics of affordance-mediating mechanisms in a creative craft process. What do *agents do with, for, and to affordances* as they engage with their ecology and how does this add to their creative pursuits? We may draw the reader's attention to these multiple “pathways” of affordance-mediated action, as creative agents notice, choose between affordances, search, explore, create, improve, modulate, turn around, reject, or nullify affordances on their interaction trajectory. Table 1 depicts a spectrum of modes of creative action, which involve different types of activity associated with an affordance; it may also furnish first steps toward a future process annotation system:

**Table 1:** Spectrum of creativity micro-functions, expressed in an affordance semantics.

**Affordance finding and exploiting**	**Affordance search and probing**	**Affordance creating and shaping**	**Affordance stimulating**	**Affordance enabling (generic)**
Rapid noticing and exploiting, i.e., using serendipitous affordances or “happy accidents”	Sensory actions that search for possibilities Manipulative actions that produce feedback on affordances by changing the constellation	Shaping actions to diversify affordances. create “springboards,” or improve a not-so-good affordance Actions that recombine known affordances into new ones Actions disclosing sequential affordances or stimulating system reactions “at a remove”	Strategic (self-) challenges or system perturbations; Gravitating toward a productive area in the action matrix, “Niche shaping” to increase the chance of some type of affordances	Actions keeping a system productive to manifest affordances

#### 4.4.1. Affordance finding and exploiting

At the junctures where creative serendipity played a role the ceramicist straightforwardly noticed a creative opportunity on the spot. On-the-spot affordance recognition is a recognized creativity mechanism (Withagen and van der Kamp, 2018; Yahklef and Rietveld, 2019) and stresses the ability to spontaneously see options or pick an uncommon action alternative that is associated with an inconspicuous or little used ecological feature. This affordance “finding” places the emphasis on the act of perceiving a creative option, and at first blush suggests “ready-to-hand” novelty. However, as we already suggested affordance finding may in fact relate to the presence of a broader creative perspective or and skilled action priors in setting up a task ecology.

#### 4.4.2. Affordance search and probing

It has been equally recognized that affordance emerge through locomotion and other actions. The ceramicist's use of hands and how she constantly probed the state of the clay bear witness to this. The specific, often subtle skills that enable this active probing can be critical for finding interesting affordances. Actions produce external changes and re-afferent feedback which suggest further affordances. As we have argued, such activity can be skillful and directed, even when it remains partly open. This category requires a consideration of the recursive process where probing actions create further feedback to reveal possible next steps. In such contexts, initially hidden *sequential affordances* (Gaver, 1991) reveal themselves only when prior affordances have been acted on. In extended tasks such as pottery imprecisely defined initial goals can hereby become increasingly disambiguated.

#### 4.4.3. Affordance creating, shaping and transforming

Less recognized in the literature is how creative affordances arise from strategically directed efforts to create an option or a class of options, hence *active affordance shaping* (Kimmel and Rogler, 2018). In our context, the moment when the vase was given its bulge as a deliberate aesthetic decision, a new field of affordances emerged in sight. We also saw moments in which serendipitous affordance finding combined with active accentuation the very next moment to work out the details of the vase's emergent silhouette. Affordance shaping is not least fundamental since many affordances “beckoning” nearby must first be improved to attain actionable quality. Ridding the clay of air bubbles is an example in which an imperfect material is improved. Perceiving potentials of such “proto-affordances” is a creative skill, both to build what is technically necessary and to transform a mundane constellation into something creatively more interesting.^5^

#### 4.4.4. Affordance stimulating and inviting

There are also slightly more open and indeterminate forms of affordance creation, which alter the probability distributions in the action system (Juarrero, 1999). Although we have no precise data on this, it is known that practitioners may stimulate a range of system reactions “at a remove”, embrace challenges, perturb the system^6^ or gravitate toward a productive area in the action matrix to stimulate novelty (Kimmel et al., 2018; Kimmel and Rogler, 2019). Moreover, strategies of *niche-shaping* (Heft, 2007; Ramstead et al., 2016) may be embraced, e.g., when using a new tool or moving to a different workshop can shift the wider *affordance landscape* (Bruineberg and Rietveld, 2014; Rietveld and Kiverstein, 2014).

#### 4.4.5. Generic affordance enabling

Expert practitioners commonly employ skilled strategies to prepare the ground, set up their action space, and keep the system manageable. Such actions can be said to be affordance-enabling. In our case, the ceramicist prepared the workshop and the clay as well as maintaining “workability” during the process by keeping qualities such as the clay's moisture around a sweet spot. Such activities create a necessary background for a whole range of further affordances, hereby setting up a space of possibilities. Although not constitutive of specific affordances, they are a pre-requisite of technical standards and all further creative processes. This expertise is about what one keeps constrained and what flexible in the system, while keeping “alive” the emergent process and poising it around states where creative things can happen, or tipping the balance in the right direction when needed. These generic skills are especially crucial in relatively open processes such as our case-study, where a constantly co-evolving ecology gives direction to the process.

To recap, we need to recognize a whole spectrum of affordance-mediated creative causalities. This includes “one-shot” creative finds, but also shaping or developing a material constellation and creating action priors, and sometimes stimulating system responses that suggest a creative path. A sole emphasis on affordance “finding” would therefore misrepresent the importance of active engagement.^7^ How a maker engages with materials supplies options that are not *per se* “just there”. In addition, experts may actively change likelihood distributions in the action system, or perform enabling background preparations that ensure that affordances of quality can emerge. This means that creativity as much depends on direct causalities as on a backdrop of indirect causalities. The latter range from workshop maintenance and material preparations, via bodily habits that provide readiness, to the subtle orientedness that manifests a crafts practitioner's particular preferences or interests. Thus, our analysis must not restrict itself to what happens in the foreground of action, but embrace a more temporally distributed perspective.

Moreover, affordance shaping, stimulating, and enabling illustrate how important directed activity over time is for creativity (Gruber, 1989). Many affordances are genuinely emergent; they cannot arise without an extended and unique interaction history. Creative opportunities thus require continuous and multi-layered *enaction* (Kimmel and Groth, 2023). They do not depend on perceptual acuity only, but equally depend on a creative perspective, proper technique, and “process savviness”, all of which conjointly make interesting affordances likely and mesh in the self-organizing causalities of continuous engagement.

## 5. Conclusion

Affordances provide a prism to nuance concerns of crafts and creativity research, when they take interest in “conversing with materials”. This reflects the growing emphasis among cognitive scientists on how creativity is grounded in materiality and embodied engagement with the ecology, rather than being a mentally encapsulated process. Our present intention was to clarify the relation of material affordances to creativity, both as regards moments of “following” the material and of “leading” it, and particularly with technical aspects in view. Specifically, we attempted to demonstrate that a comprehensive “*in-vivo*” process analysis with a high zoom factor can advance the debate and elucidate the multiple roles that affordances play in the texture of an aesthetic and creative process.

The case-study unpacked how affordances mediated the pivotal decisions that “route” a unique crafts process across its different stages. As a vase was created, each affordance-based action begets the next in a path-dependent fashion. By acting on momentary affordances the crafts practitioner generated an evolving *affordance field* in which she responded to, yet also re-shaped the material ecology. The aesthetically focused second half of the case-study broadly confirm Schön's (1991) views and Malafouris (2013), *material engagement theory* which would predict that affordances emerge by exploring and interacting with the material's possibilities. This notwithstanding, the ceramicist also constrained the emergent process sufficiently so actions would cohere across time, which indicates a highly selective relationship to possible material affordances. Within these constraints, we have seen the dynamics move between moments of serendipity, active shaping, creating and refining inspirations, working out prior ideas, setting next objectives, and subsequent problem solving. The expert practitioner thus employed a variety of means for creativity within one project, an insight which relates to our next point.

An important theoretical objective was to distinguish ways in which affordances mediate creativity. Some moments relate to direct “finds” suggesting creative options on the spot. Thus, serendipity accounted for about half of creative routing points during the creative part of the vase making. However, we widened this picture by pointing to a set of less discussed mechanisms: how mundane options are developed into creative options, how practitioners shape the general probability space, and how they set up an enabling background for creativity. From this wider perspective, the ability to bring forth novel, yet also fitting affordances through material engagement reflects a mix of constrained exploration, the ability to perceive and harness chance occurrences to one's needs, as well as creative orientedness and imaginative potential.

By further implication, affordance theorist should not think of crafts creativity as mere *resonance* with the ecology, a view that sets a too passive emphasis. Many of our observations reflect a great deal of creative autonomy. It is important to realize that how a person sets up and orchestrates the extended material engagement over time inherently shapes whether and which affordances emerge. This is why we stressed that even serendipitous affordance “finds” depend not only on perceptual skill. They causally emerge from skillful prior engagement and a creative perspective which guides the active development of what was “found”.

Applying affordances to creative crafts (and similarly visual art, invention, or science) requires a great deal of holism in the analysis. Expert practitioners orient toward affordances spanning multiple timescales. Attending to affordances that evolve slowly in the background is just as important as attending to faster changing ones. What is more, affordance related criteria that are processual and technical, as well as such that are functional, aesthetic and creative always remain co-present, orientations that may sometimes require compromise or prioritizing.

Another critical insight was that a creative process depends less on individual affordances (or local actions) than on how the whole fits together. Affordances indicating one aesthetic feature are typically evaluated in the light of existing or anticipated further features once a creative project is moving in a certain direction. Sensitivity to affordances is thus integrally orchestrated. It would be handy to explain a creative process in terms of one central affordance, but that is seldom possible. We must instead consider how a perceptually grounded imagination connects individual affordances into something larger. This is needed to ensure that momentary aesthetic choices cohere with projected later ones, all within holistic awareness of the task.

In the end, any creative process is sustained by its surrounding technical best practices, which are essential for creative opportunities to arise in the first place. Creativity subsists in extended and integral activities of meaning making. Thus, we must not privilege “creative” affordances in the analysis, but should contextualize the creative aspect more broadly. In other words, we should study *affordances in creative activities* rather than just studying “creative affordances”. After all, the unit of analysis is a spatio-temporally extended ecology of practice, and its unique transformational potentials. This emphasis casts a critical light not only on the tendency in cognitive research to talk about “canonical affordances”, but also the frequent practice of de-contextualizing affordances from their highly situated task contexts and task histories.

## Data availability statement

The original contributions presented in the study are included in the article, further inquiries can be directed to the corresponding author.

## Ethics statement

Ethical review and approval was not required for the study on human participants in accordance with the local legislation and institutional requirements. The patients/participants provided their written informed consent to participate in this study. Written informed consent was obtained from the individual(s) for the publication of any potentially identifiable images or data included in this article.

## Author contributions

MK wrote and prepared the manuscript. CG provided feedback and small additions as well as edits to the manuscript. The data collection was jointly organized, conducted, and analyzed by MK and CG. All authors contributed to the article and approved the submitted version.

## Footnotes

^1^In addition, creativity is no longer seen as a solitary practice; it involves collaborative aspects (Sawyer, 2003, 2006) and involves socially distributed processes (Sawyer and DeZutter, 2009). Accordingly, these views question *methodological individualism* and make wider systems the unit of analysis.

^2^These views define creativity as a function of coupling dynamics and stress embodied engaging. In this context, dynamic systems approaches have claimed that the relationship between a person and the environment can self-organize in creative ways (Walton et al., 2015, 2017; Orth et al., 2017) when the systemic context is right, e.g., through constraints that narrow down the possibility space and encourage particular explorations (Hristovski et al., 2011; Torrents et al., 2021).

^3^This approach is further supported by ethnographic sketches of affordance-mediated architectural and design activity (Rietveld and Brouwers, 2017) and attempts to highlight that the imagination has properties of affordances of the larger scale (van Dijk and Rietveld, 2017, 2018).

^4^This type of creative decision comes about through emergent dynamics in the system of maker and material when micro-technical procedures produce ripple effects, which suggest a new large-scale aesthetic direction.

^5^Data we collected on other crafts processes suggests that transforming mundane features to provide them with interesting new functions is not infrequent, as is inventing non-standard action responses to conventional affordances.

^6^This may involve embodied creative *problem finding*, the counterpart to *problem solving* (Runco, 1994), when some introduces a constraint or difficulty actively because this can in turn stimulate the exploration or creation of alternative affordances. Even momentarily dis-afforded situations may be created on purpose for new affordances to emerge down the line, as studies on dance show (Kimmel and Hristova, 2021).

^7^Even moments of creative serendipity should not be understood as mere affordance “finds”. For happy accidents that lend themselves to creative action a backdrop of suitable orientedness may be needed in the first place.
